# Ultrafast PCR Detection of COVID-19 by Using a Microfluidic Chip-Based System

**DOI:** 10.3390/bioengineering9100548

**Published:** 2022-10-13

**Authors:** Xiaojing Chen, Yiteng Liu, Xuan Zhan, Yibo Gao, Zhongyi Sun, Weijia Wen, Weidong Zheng

**Affiliations:** 1 Department of Laboratory Medicine, Shenzhen University General Hospital, Shenzhen 518000, China; 2 Interdisciplinary Programs Office, Hongkong University of Science and Technology, Hong Kong SAR 999077, China; 3 Department of Physics, Hongkong University of Science and Technology, Hong Kong SAR 999077, China; 4 Department of Urology, Shenzhen University General Hospital, Shenzhen 518000, China; 5 HKUST Shenzhen-Hong Kong Collaborative Innovation Research Institute, Shenzhen 518000, China

**Keywords:** microchip-based system, polymerase chain reaction, microfluidics, COVID-19, point-of-care test

## Abstract

With the evolution of the pandemic caused by the Coronavirus disease of 2019 (COVID-19), reverse transcriptase-polymerase chain reactions (RT-PCR) have invariably been a golden standard in clinical diagnosis. Nevertheless, the traditional polymerase chain reaction (PCR) is not feasible for field application due to its drawbacks, such as time-consuming and laboratory-based dependence. To overcome these challenges, a microchip-based ultrafast PCR system called SWM-02 was proposed to make PCR assay in a rapid, portable, and low-cost strategy. This novel platform can perform 6-sample detection per run using multiple fluorescent channels and complete an ultrafast COVID-19 RT-PCR test within 40 min. Here, we evaluated the performance of the microdevice using the gradient-diluted COVID-19 reference samples and commercial PCR kit and determined its limit-of-detection (LoD) as 500 copies/mL, whose variation coefficients for the nucleocapsid (N) gene and open reading frame 1 ab region (ORF1ab) gene are 1.427% and 0.7872%, respectively. The system also revealed an excellent linear correlation between cycle threshold (Ct) values and dilution factors (R^2^ > 0.99). Additionally, we successfully detected the target RNAs and internal gene in the clinical samples by fast PCR, which shows strong consistency with conventional PCR protocol. Hence, with compact dimension, user-friendly design, and fast processing time, SWM-02 has the capability of offering timely and sensitive on-site molecular diagnosis for prevention and control of pathogen transmission.

## 1. Introduction

Coronavirus disease 2019 (COVID-19) is a pneumonia caused by Severe Acute Respiratory Syndrome Coronavirus 2 (SARS-CoV-2). In 2020, COVID-19 rapidly evolved into a global pandemic and took many lives, resulting in worldwide economic and social destruction [1]. Presently, medical institutions use various diagnostic methods, including ELISA (enzyme-linked immunosorbent assay) [2], medical imageology [3], and molecular diagnostic for COVID-19 detection [4]. Considered as the acknowledged detection standard of pathogenic microbes [5], reverse transcriptase-polymerase chain reaction- (RT-PCR) based assays can amplify the DNA/RNA under variable temperature conditions, which can determine the symptomatic or asymptomatic carrier of COVID-19 with high sensitivity and specificity [6,7]. Polymerase chain reaction (PCR) technology offers an effective way to screen the sample with low viral concentration at an early stage [8,9], whereas limitations, such as tedious turnaround time and expensive cost, essentially block its further application in pandemic prevention [10]. For instance, using a Peltier-based heater, conventional PCR devices perform nucleic acid amplification in plastic tubes for several hours due to their low thermal transmission rate [11]. Besides, conventional PCR instruments must be set up in clean rooms that require airflow control and isolation design [12].

According to the World Health Organization (WHO) guidelines for COVID-19 prevention, timely detection, and isolation of infected patients can effectively interrupt the novel coronavirus transmission [13]. Presently, lacking of specialized facilities, collected samples from the respiratory tract need to be transported to the central hospital under low-temperature conditions [14]. Long-distance transport between the lab and epidemic area not only delay testing reports but also raise the risk of nucleic acid degradation that may raise false-negative results [15,16]. Point-of-care testing (POCT), with superior accessibility and timeliness, refers to the diagnostic tests performed near the patient, including blood glucose monitoring devices, urine test strips, pregnancy self-test strips, etc. [17,18,19,20,21]. Microfluidic technique has been used in the development of field PCR instruments, which enables miniaturized design and rapid thermal conductivity since its high surface-to-volume ratio in microscale [22]. The microfluidics networks like microchambers and microchannels also can significantly reduce the reagents consumption and offer an enclosed environment for a nucleic acid test (NAT) [23]. Therefore, during the outbreak of infectious diseases like COVID-19, a rapid and economical Microchip-based PCR system with a fully portable and sealed reaction chamber design can greatly relieve the diagnosis burden in the resource-limited area.

In order to overcome the limitations of conventional PCR technology, microfluidic chip-based PCR has been studied in recent decades, and many scientists focused on how to condense the whole process of PCR with functional micro-components [24,25,26,27]. Chen and Mauk et al. mixed up extracted sample and reaction reagents by valve and conducted the 40–45 thermal cycles using a microheater for DNA amplification. The PCR cartridge was able to control the liquid flow by actuation forces and condense the assay process [23,28]. However, this strategy is still staying at the proof-of-concept stage and only one sample could be tested within a single round due to its high integration. Xing and Liu et al. present isothermal PCR assays with a commercial analyzer (RTisochip™-W) for multiplex detection of respiratory viruses including COVID-19, which can handle 16 samples in a single run within 90 min [29]. The isothermal NAT method could avoid temperature variation demand and reduce turnaround time compared with traditional PCR instruments, but nonspecific amplification increasing the risk of false-positive result easily occurs, and its detection time can be further condensed as a microchip-based device [30,31,32].

Although many strategies have successfully conducted the quantitative or qualitative trials for nucleic acid detection with a microfluidic system, the POCT medical devices used in COVID-19 screening scenarios are facing several challenges: (1) portability, compared with the lab-based instruments, POCT devices need to minimize its dimension and power demand for on-site detection since many areas particularly in the remote region are lack of sufficient experimental conditions [33]. (2) Detection efficiency. Considering the urgent demand for suspected cases screening, the detection time should be shortened as much as possible facing extensive sample collection. Furthermore, high-throughput diagnostic assays can efficiently identify SARS-CoV-2 infected patients with similar symptoms among the other respiratory viruses [34]. (3) User-friendly design. Field level tests require to reduce the operational difficulties and workload for users. Integrated and self-driven design in the POCT device can simplify tedious pipetting steps as well as the risk of sample cross-contamination [35]. Thus, sensitive field detection of COVID-19 can benefit the epidemic control and enhancement of local detectability because of its quick response to emergency and high mobility.

In this work, we evaluated an ultrafast COVID-19 diagnostic platform, the SWM-02 microfluidic PCR analyzer, which contains silicon-based microchips, a compact optical module, and a low power consumption microheater. Several 12μL chambers are formed in this microchip by etching technology and they can perform multiple NATs simultaneously ensuring high throughput of the microdevice. Silicon is used as substrate material of the microchip and microheater, which can significantly save the assay time and cost of massive production [36,37]. Besides, a fully sealed design of microchip is available for field detection and lowers the risk of aerosol contamination during the amplification process. There are three simple and user-friendly steps for startup PCR in the SWM-02; (1) load the PCR mixture into the microchip without external force; (2) insert the sealed microchip in the machine; (3) setup and run the PCR protocols. Merits of microfluidics facilitate the SWM-02 to perform bioassays in a rapid, convenient, and cost-effective way. Here, we assessed the heating performance of the microheater and then demonstrated whether this novel microdevice provides a feasible approach for ultrafast PCR detection of SARS-CoV-2.

## 2. Materials and Methods

### 2.1. SWM-02 Unltrafast PCR Microchip-Based Platform

**Figure 1:**
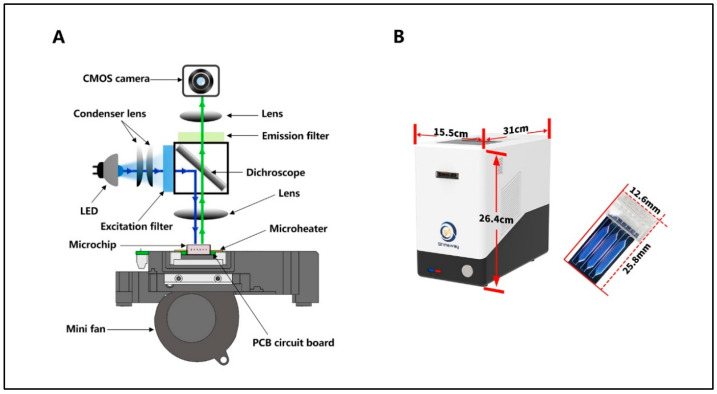
(**A**) Design and configuration diagram of the multiple fluorescent collection module. (**B**) Outlook and dimension of Real-time PCR Nucleic Acid Detection System SWM-02 and microchip BS-C3-12.

As shown in Figure 1A, the SWM-02 microchip-based platform (Shineway, Shenzhen, China) mainly consists of a temperature control module and a fluorescent collection module. Particularly, the microheater is welded on the PCB circuit board and contacted tightly with the microchip for rapid heating. A high-speed fan is also fixed behind the microheater to lower the temperature. Upon the microchip, the optical module involves a light-emitting diode (LED) light with three peak wavelengths (475~480 nm, 535~540 nm and 620~625 nm), a dichroic mirror, filters with multiple pass bands, a set of lenses, and a complementary Metal Oxide Semiconductor (CMOS) camera. Figure 1A also illustrates the process of the multi-fluorescent collection. Firstly, the condensed excitation light (blue line) is filtered and then reflected by the dichroscope toward the PCR microchip. Secondly, as the nucleic acid amplification process, accumulated fluorescence in the PCR mixture is excited and the emission light (green line) would pass the dichroscope selectively. The dichroscope is set up in the optical module at 45° to reflect and transmit excitation and emission light, respectively. Finally, emission light emitted from the fluorescent signal is filtered by an emission filter for noisy elimination and reaches the CMOS camera.

In the Figure 1B, the dimension of the SWM-02 microfluidic analyzer is 31 cm × 15.5 cm × 26.4 cm (length × width × height) with a weight of 3.5 kg. BS-C3-12 microchip (Shineway, Shenzhen, China), composed of silicon and glass layers, is fabricated by standard micro-electromechanical systems (MEMS) processes. Using photolithograph and dry etching technologies, microfluidic networks are developed on a silicon substrate. The microchamber is formed with a volume of 12 μL after a layer of transparent glass is boned on the silicon surface. Especially, the plastic cap is designed to inserted into the top of the microchip, which provides biological reaction with a fully enclosed environment in case of cross-contamination. This microsystem can perform six nucleic acid tests simultaneously using two microchips.

### 2.2. Control Scheme of the SWM-02

**Figure 2:**
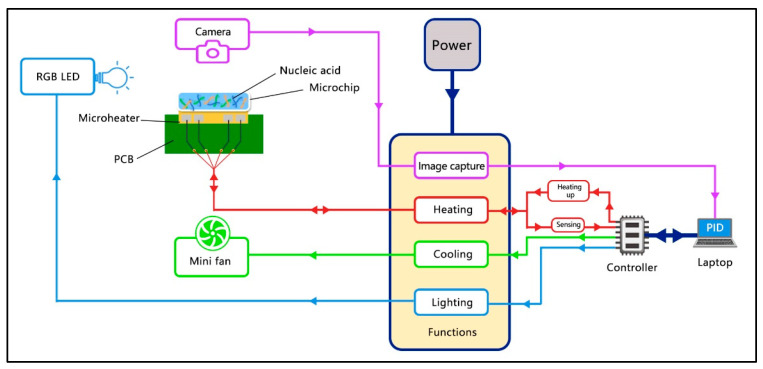
A general control scheme of the SWM-02 microfluidic PCR control system.

As shown in Figure 2, the multifunctional implementation of SWM-02 is based on the upper and lower control with python code-based software. A laptop and the controller serve as upper and salve machines, respectively. For more details, once we set up the thermal procedure in the software, the real-time temperature value is detected by the temperature sensor inside the microheater and collected by a controller. The temperature data is imported through communication interface transmission to the laptop computer for calculation and comparison with the setting value. Afterwards, an output command, including heating duration and cycle number, is transmitted to the controller to control the power distribution. As a result, the microheater and cooling fan would run or stop following the controller’s order, which makes the real-time temperature reach the target value. Similarly, the RGB (red, blue, and green), LED, and CMOS camera were operated during the signal collecting process according to the user setting. After completion of the reaction protocol, the data, such as temperature profile, fluorescence intensity and pictures at the endpoint of every cycle, would be saved and analyzed on the laptop.

### 2.3. Materials and Sample Pretreatment

In this validation study, we chose pseudovirus nucleic acid reference material (Batch number: 2022011, open reading frame 1 ab region (ORF1ab) gene:2.0 × 10^2^ copy/μL, nucleocapsid (N) gene:2.0 × 10^2^ copy/μL, GBW(E)091132, Guangzhou Bangdesheng Biotechnology Co., Ltd., Guangzhou, China) obtained from NMIC (National Institute of Metrology, Beijing, China) as the positive sample for repeatability and detection limit studies of the ultrafast PCR platform. Following the user manual, the synthetic samples were purified by MagaBio plus Virus DNA/RNA Purification Kit III (BSC86S1E, BIOER, Hangzhou, China) and GenePure Pro Nucleic Acid Purification System (NPA-32P, BIOER, Hangzhou, China). Thereafter, serial dilution of the reference DNA/RNA is set up with different ratios (1:20, 1:200, 1:400, 1:1000), which are equal to the concentrations of the target genes (ORF1ab and N gene) of 10,000, 1000, 500, and 200 copies per mL, respectively.

As for clinical evaluation, nasopharyngeal swabs in virus preservation material (VPM) were collected from confirmed cases with COVID-19 (*n* =24) and healthy people (*n* = 91). Exemption of informed consent is appropriate for this study since we used in vitro residual samples from clinical diagnosis and didn’t cause new risks such as wrong treatment instruction. All collected samples were anonymized and a waiver of informed consent was obtained. After sample collection, VPM with throat swabs were transported and stored at −80 °C. A 300 μL immersion solution was prepared for nucleic acid extraction using a Nucleic Acid Isolation Kit (DA0602, Daan Gene, Guangzhou, China) and Smart 32 Nucleic Acid Extraction Instrument (Daan Gene, Guangzhou, China) in accordance with the manual protocol. The extracted samples were transferred to a 1.5 mL nuclease-free centrifuge tube and stored at −80 °C for later use. The above operations are performed in the Department of Laboratory Medicine, Shenzhen University General Hospital (Shenzhen, China), and we adopted extracted sample for validating the clinical performance of the SWM-02 microfluidic PCR analyzer with a commercial PCR machine (LightCycler 480, Roche, Basel, Switzerland).

A novel coronavirus 2019-nCoV nucleic acid detection kit (Fluorescent PCR method) purchased from Daan Gene Co., Ltd. (DA0990, Batch number: 2022399) was used for two experiments mentioned before. 6-carboxyfluorescein (FAM), Hexachloro-fluorescein (HEX), and Cyanine 5 (Cy5) represent the fluorescent dyes used in the RT-PCR. Based on one-step RT-PCR technology, this commercial kit selects the ORF1ab and N gene regions of SARS-CoV-2 as targeted sequences, and designs two sets of specific primers and fluorescent probes (ORF1ab gene is labelled with FAM and N gene with HEX) for the COVID-19 detection. Except for two target genes, the reference gene ribonuclease P (RNase P) is labelled by Cy5 is tested for sample quality assessment.

### 2.4. Reagent Preparation and PCR Condition

The operation protocol of the SWM-02 is divided into three steps: (1) mix the extracted sample with PCR reagents; (2) load the mixture into the microchip and seal the microchip; (3) insert the sealed microchip into the device and set up PCR condition. Users are required to be familiar with pipetting and mixing operations in the biomolecular experiment. According to the user manual of the PCR kit, for the SWM-02 device and LightCycler 480, the total reaction volume is 25 μL which consists of 17 μL 2019-nCoV PCR solution A, 3 μL 2019-nCoV PCR solution B, and 5 μL Extracted Sample. After blending the reaction mixture, 12 μL of total solution would be used for the on-chip test. There is no external force demand for the SWM-02 platform during the sample loading process because the solution in the pipette tip can be injected into the microwell automatically once it touches with input channel of the microchip due to the capillary effect. Then, we set up the following conditions for thermal cycling of COVID-19 RNA standard samples and clinical samples: (1) reverse transcription: 50 °C for 2 min; (2) pre-cycle: 95 °C for 2 min; (3) 10 thermal cycles: 95 °C for 5 s and 60 °C for 35 s; (4) 32 thermal cycles: 95 °C for 5 s and 60 °C for 35 s, fluorescent collection. The PCR result is determined by referring to the user manual of the PCR kits.

## 3. Results and Discussion

### 3.1. Thermal Properties of Ultrafast PCR Microdevice

**Figure 3:**
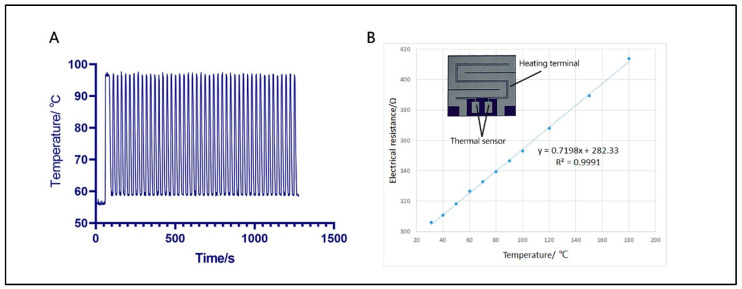
Thermal performance of the microheater. (**A**) Temperature profile in thermal cycling. (**B**) The linear relationship between electrical resistance and real-time temperature (R^2^ = 0.999) and patterned microheater with heating and sensor terminal.

Based on our previous teamwork, a silicon-based microheater was successfully developed for rapid digital PCR and finished 45 cycles in 36 min [38]. In this ultrafast PCR platform, silicon was still chosen as the substrate material of the microheater. The heating performance of SWM-02 has been improved by expanding the heating area and optimizing the voltage between two platinum electrodes. As shown in Figure 3A, the highest heating rate can reach 35 °C/s, and a standard RT-PCR procedure was completed in 25 min (including the reverse transcription process). The total PCR time could be significantly lowered and increase the working efficiency for on-site detection. In Figure 3B, the microheater with platinum (Pt) coating consists of a heating terminal and a thermal sensor. Pt shows a good linear correlation between sensor terminal resistance and temperature (R^2^ = 0.9991). This indicates that SWM-02 can provide accurate temperature control for biochemical reaction. Close contacting with the silicon microfluidic chips, the microheater could recognize a quick temperature ramping as high heat conductivity of silicon. Moreover, the mass of microheater is much lighter than Peltier semiconductor-based heating block, which could not only improve the portability of the machine, but also reduce the power consumption. Therefore, in contrast to conventional heating methodology, microheater fabricated by silicon and platinum is more feasible for outdoor operation, and fulfills the requirements of point-of-care detection.

### 3.2. Limit-of-Detection and Reproducibility

**Figure 4:**
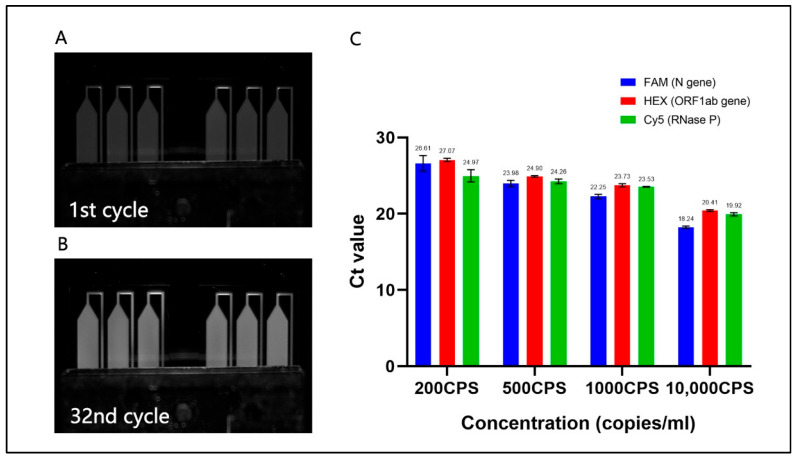
Fluorescent photos of two 3-well microchips (1000 copies/mL (CPS)) captured by CMOS camera (FAM) at the endpoint of the 1st cycle (**A**) and the 32nd cycle (**B**). (**C**)Average Ct values of N gene, ORF1ab gene, and RNase P. Added concentration of N gene and ORF1ab gene are 10,000, 1000, 500, and 200 CPS per mL. Correspondingly, the dilution factors of reference gene (RNase P) are 20, 200, 400, and 1000. Error bars represent the standard deviation of the 3 replicates.

As shown in Figure 4A,B, there was a significant difference in fluorescent intensity between the beginning and last cycle of reaction using 1000 copies/mL reference sample, which means that PCR assay has been successfully performed in six microchambers. In the darkroom, the CMOS camera collects pictures at the grey level, and white brightness appearing in microchambers would be enhanced as the increase of marked amplicon. Figure 4C summarized Ct values of PCR with plasmid samples ranging from 200 copies/mL (CPS) to 10,000 CPS and Ct values obtained in three channels showed a declining tendency as the increase of sample concentration. Each diluted sample is tested three times for amplification of the target gene. Among 200 CPS of three specific sequences (N genes, ORF1ab and RNase P), the Ct values of the ORF1ab gene reached the highest 27.07, indicating 200 copies/mL of COVID-19 standard sample can be detected by this novel platform. The amount of RNase P is estimated by the approximations of the other two genes because its initial is unknown. Apart from 200 CPS, the short error bars of the average Ct value represented the high repeatability of the rapid PCR device.

**Figure 5:**
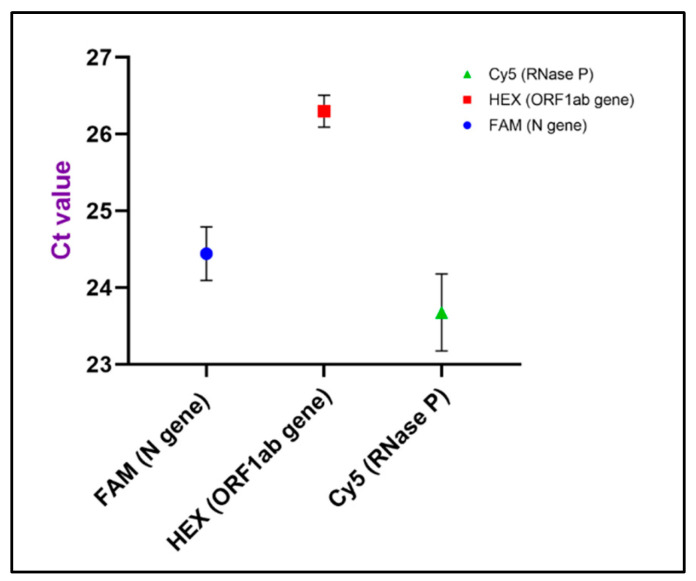
Average Ct values of the 500 CPS standard samples at six replicates using the ultrafast PCR platform. Error bars represent the standard deviation of the 6 replicates.

Based on the above results, we chose 500 CPS reference sample to evaluate the LoD of the microdevice. Reproducibility of the detection limit was verified by testing 6 repeats at target genes and internal genes. As shown in Figure 5, the Ct values of the N gene, ORF1ab gene and RNase P were 24.44, 26.3 and 23.68, respectively, and two target genes displayed relatively short error bars. From Table 1 we can see that coefficients of variation for three genes were all less than 3.0%, which indicated that the microsystem could provide highly reproducible results using 500 CPS sample. The Ct values of three genes fulfilled the positive diagnosis criteria of PCR reagent kit and all the replicates were identified as positive results. Theoretically, the detection limit of the ultrafast PCR device is at least 500 copies/mL. Among the approved nucleic acid test products by WHO Emergency Use Listing (EUL), the desirable LoD is 100 copies/mL obtained by conventional quantitative PCR (qPCR) protocol [39]. Although LoD of the ultrafast PCR mode cannot achieve the desired level, 500 copies/mL is satisfying for the technical requirement of the rapid PCR kit used in this study [40]. Compared with traditional protocol, the initial concentration of template DNA used on the SWM-02 scheme is lower since its duration of reverse transcription (RT) is 2 min (20 min for traditional RT-PCR) and less extracted sample consumption. Meanwhile, this reagent-saving PCR method with acceptable LoD not only could reduce testing costs, but also fulfil the demand for early on-site COVID-19 screening. Hence, with high sensitivity and reproducibility, this microchip-based device has the capability of offering the real-time PCR test for COVID-19 detection in time.

### 3.3. Linear Correlation

**Figure 6:**
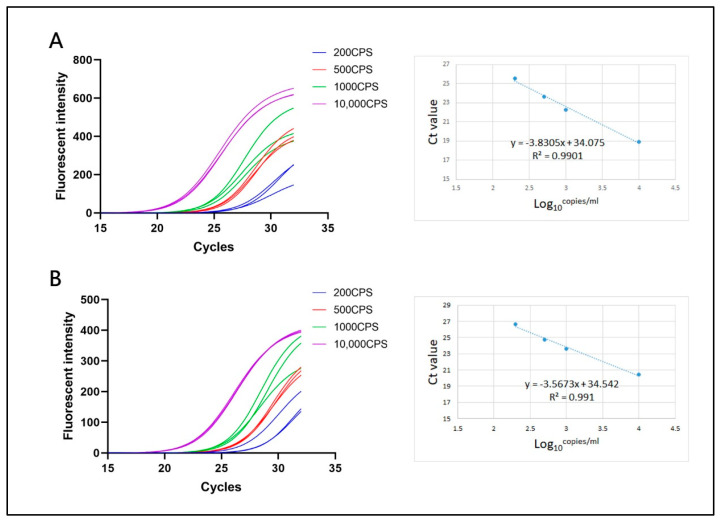
After a standard 42-cycle (including 10 pre-cycles without fluorescence collection) rapid RT-PCR, the fluorescence curves and the linear relationship between Ct value and logarithmic sample concentration (Log10) of N gene (**A**) and ORF1ab gene (**B**). The standard samples of SARS-CoV-2 are derived from 10,000, 1000, 500, and 200 copies/mL and perform 3 replicates for each concentration.

In Figure 6, the amplification curve of target genes revealed an exponential increase of fluorescence intensity using reference samples derived from different diluted factors. As for high concentration samples (1000 copied/mL), the amplification curve reached a plateau phase with average Ct value of 18.92 (N gene) and 20.41(ORF1ab gene). Although other sample concentrations did not show the distinct steady stage at the endpoint of reaction, it can diagnose positive results based on three repeated tests. This atypical amplification curve would be compromised for fast on-site microbial detection, since the Ct value is identified in the microdevice when the fluorescent intensity reaches the threshold level. On the right side of Figure 6, it demonstrated a good linear correlation between Ct values and sample concentration in all diluted factors. The linear coefficient R^2^ of N gene (A) and ORF1ab gene (B) were 0.990 and 0.991, respectively. It is noted that a certain amount of two target genes can be potentially determined by a standard curve using this ultrafast PCR platform.

### 3.4. Clinical Evaluation between On-Chip PCR and Conventional qPCR

**Figure 7:**
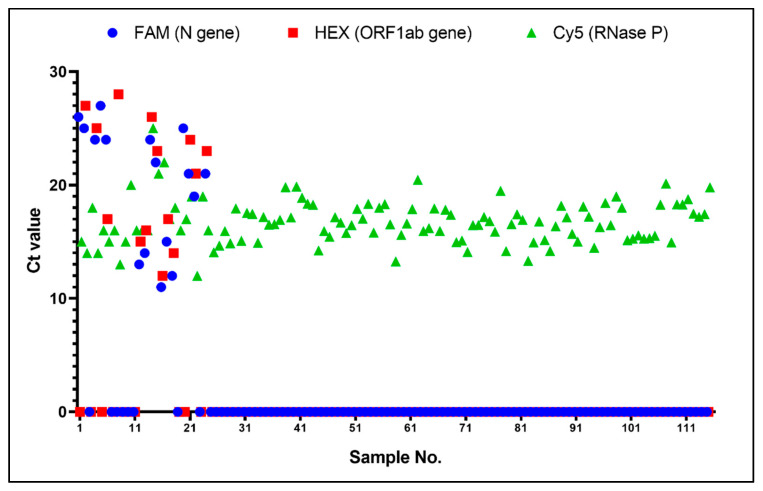
Ct values of 1~115 clinical samples using SWM-02 ultrafast PCR platform (N gene, ORF1ab gene, and RNase P).

Extracted nucleic acid samples involving 24 positive and 91 negative cases were collected from the hospital for clinical validation of the SWM-02 ultrafast platform. All clinical samples were previously determined employing real-time PCR by LightCycler480. As shown in Figure 7, Ct values ranging from 11 to 28 of specific target genes were observed for positive samples and there was no obvious amplification signal for negative samples. Additionally, the RNase P gene of every nasopharyngeal sample could be detected with certain Ct values indicating that sampling was effective in this study. As for coincidence rate analysis, 17 and 18 out of 24 positive cases were conformably detected in SWM-02 (70.83%) and LightCycler480 (75%), respectively. While all the negative cases can be correctly distinguished in both instruments (100% specificity). Specifically, both devices cannot identify the No. 3, 7, 10, 11, 19, and 23 positive cases because some patients with COVID-19 were confirmed by clinical symptoms and imageology, and their viral RNA concentrations were lower than the detection limit of the PCR assay. It is worth noting that SWM-02 showed different detection result of the No.6 sample with the reference device, demonstrating a gap in clinical LoD between the two devices. Compared with the LightCycler480, the microchip-based platform used half of the sample volume, so lower RNA template concentration may cause this deviation. Kappa analysis was performed to verify the clinical performance of the SWM-02 platform with traditional PCR machine. The Kappa value was 0.965 (higher than 0.90) [41], which revealed perfect agreement between the two devices (Table 2). Thus, it is worth noting that the rapid PCR tests conducted on the SWM-02 can correctly detect COVID-19 in clinical application in 40 min rather than RT-PCR on the benchtop machine (normally 2–3 h).

## 4. Conclusions

In summary, we validated a novel COVID-19 diagnostic platform, which contains a multiple fluorescence detection module, silicon-based microchip, and low power consumption microheater. Microchips use silicon and glass as raw materials so that it can further reduce the cost of manufacture and enhance heat conductivity between microchip and microheater. Thus, SWM-02 can significantly condense the total time of PCR and increase the feasibility of the PCR technique for on-site detection. In contrast to other PCR microsystems, with high detection throughput, this microdevice can achieve a standard RT-PCR for COVID-19 detection within 40 min. Then, its detection limit was confirmed using 500 copies/mL standard reference sample and linear coefficients of two target genes between sample concentration and Ct values were above 0.99, demonstrating that quantitative assay of viral RNA is possibly realized in the ultrafast mode. Additionally, the SWM-02 platform can provide a highly comparable diagnostic result with traditional PCR instruments in clinical evaluation. Hence, with the capability of fully portable size, saving time, low cost, and functional integration, this microfluidic equipment fulfills the requirement of the COVID-19 field test, which facilitates molecular diagnosis in pandemic prevention and healthcare management.

## Figures and Tables

**Table 1:** Variation of Ct values for PCR assays in 500 CPS standard samples using the SWM-02 ultrafast PCR platform (*n* = 6).

No	FAM (N Gene)	HEX (ORF1ab Gene)	Cy5 (RNase P)
1	24.59	26.48	23.9
2	24.03	26.19	23.92
3	24.77	26.28	23.84
4	24.06	26.57	22.66
5	24.84	25.99	23.8
6	24.37	26.28	23.94
Mean	24.44	26.3	23.68
SD	0.3489	0.207	0.5008
CV	1.427%	0.7872%	2.115%

**Table 2:** Crosstabulation of detection result between SWM-02 and LightCycler480 and Symmetric Measures of Kappa analysis.

Count		LightCycler480		Total	
		Negative	Positive		
SWM-02	Negative	98	1	99	
	Positive	0	16	16	
Total		98	17	115	
		Value	Asymptotic Standard Error ^1^	Approximate T ^2^	Approximate Significance
Measure of AgreementKappa		0.965	0.035	10.351	0
N of Valid Cases		115			

^1^ Not assuming the null hypothesis. ^2^ Using the asymptotic standard error assuming the null hypothesis.

## Data Availability

Not applicable.
